# Reducing the expression of the Numb adaptor protein in neurons increases the searching behavior of *Drosophila* larvae

**DOI:** 10.17912/micropub.biology.000426

**Published:** 2021-07-26

**Authors:** Andrew Galbraith, Samuel Leone, Katherine Stuart, Josie Emery, M. Katie Renkemeyer, Nicholas Pritchett, Lauren Galbraith, Haley Stuckmeyer, Brett Berke

**Affiliations:** 1 Department of Biology, Truman State University, Kirksville, MO USA

## Abstract

*Drosophila* larval crawling is easily-observable and relatively stereotyped. Crawling consists of linear locomotion interrupted by periods when the larvae pause, swing their heads, and change direction (a ‘search’). Here we identify Numb, a peripheral membrane adaptor protein, as an important regulator of searching behavior. When Numb RNAi transgenes were expressed in all neurons, searching frequency increased while linear movement appeared normal. Numb’s role in suppressing searching behavior was verified by rescuing this phenotype with a Numb homologue from mice. Such behavioral specificity suggests that further analysis of searching might help identify additional, evolutionarily-conserved interactors of the Numb protein.

**Figure 1. RNAi-mediated knock down (KD) of Drosophila Numb (d-numb) in all neurons increases irregular larval crawling, which can be rescued by co-expressing an RNAi-insensitive Numb homologue from mice:**
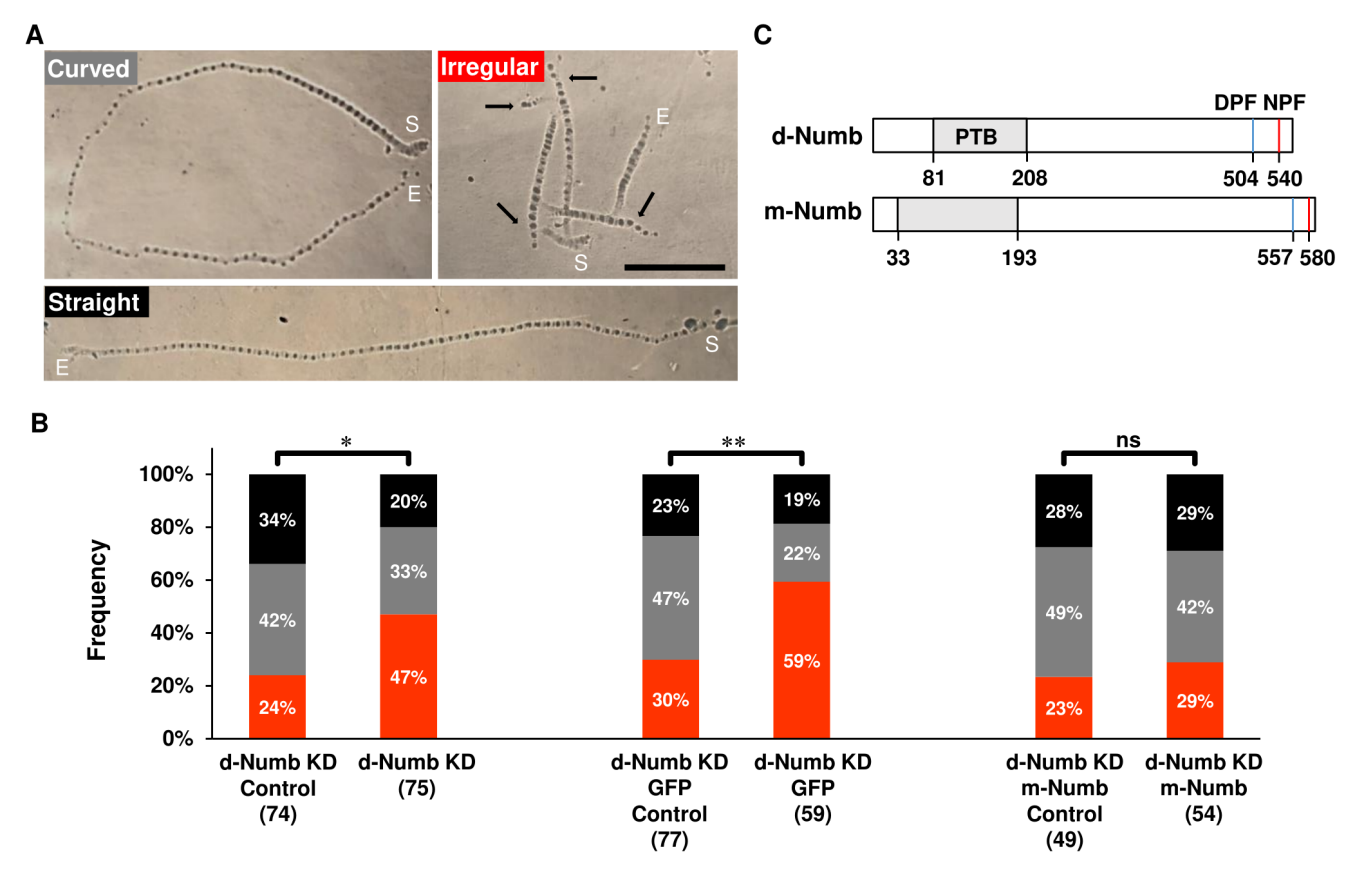
**(A)** Wandering-stage larvae show three different path types (straight, curved, and irregular) based on the pattern of their mouth-hook imprints. Start of the path, S. End of the path, E. Irregular paths were defined as trajectories with 3 or more ‘searches’, indicated by arrows. See the Methods for the definition of a search and for parameters defining each path type. **(B)** The proportion of irregular crawling paths is increased by NumbKD in all neurons relative to its uninduced control (left two bars; genotype: w ; ; ELAV-GS-GAL4 / UAS-dNumb^ds 5,10^). The condition and number of larvae tested is indicated below each bar. Expression of GFP, in addition to the RNAi, does not lessen this locomotor phenotype (middle two bars; genotype: w ; ; ELAV-GS-GAL4 / UAS-CD8-GFP). In contrast, the increased search behavior that led to the prevalence of irregular crawling paths was rescued by co-expressing a Numb homologue from mouse (mNumb) that is insensitive to the RNAi transgene (right two bars; w ; UAS-myc-mouse-Numb#4 / + ; ELAV-GS-GAL4 / UAS-dNumb^ds 5,10^). * (p<0.05), ** (p<0.005), and ns (not significant). **(C)** Numb from *Drosophila* and mouse are homologues with a high degree of identity and each share PTB, NPF, and DPF domains.

## Description

Numb was first identified as an adaptor protein required for *Drosophila melanogaster* sensory organ formation (Uemura *et al.*, 1989). Over time, Numb and its binding partners have been implicated in a variety of cellular processes, including neurogenesis (Zhong *et al.*, 1996), chemotaxis (P. Zhou *et al.*, 2011), axon outgrowth (Huang *et al.*, 2005), synapse formation (L. Zhou *et al.*, 2015), social behavior (Wang *et al.*, 2019), and cancer (Pece *et al.*, 2011).

In *Drosophila*,loss of function Numb mutations alter the development of both central and peripheral neurons, including the networks that mediate larval crawling behavior (Spana *et al.*, 1995, 1995; Tang *et al.*, 2005; Vervoort *et al.*, 1997). Larvae crawl through peristaltic waves of posterior to anterior body wall contractions that hydrostatically push the head forward to allow the animal’s mouth hooks to anchor at a new location on the substrate. Forward (and backward) crawling requires the activity of central pattern generators within the larval ventral ganglia (Clark *et al.*, 2018; Fox *et al.*, 2006). These networks set the rate and intensity of body wall contractions (Wang *et al.*, 1997, 2002), integrating sensory feedback from multidendritic cells and chordotonal organs in the larval body wall (Hughes & Thomas, 2007; Ohyama *et al.*, 2013; Song *et al.*, 2007). Periodically, larvae will pause, swing their head, and begin crawling in a new direction (called here, a ‘search’). The mixture of linear crawling and searching behavior leads to several distinct crawling trajectories that can be identified by video (Heckscher *et al.*, 2012; Wang *et al.*, 2002) and by the pattern of mouth hook imprints when larvae crawl on soft substrates (Fig. 1A). Larval crawling has been extensively studied with genetic tools. However, most mutational and neural circuit dissection techniques have uncovered the biology of linear crawling, with fewer genes and cells being implicated in searching behavior.

Here, we show that Numb regulates the searching behavior of wandering-stage larvae. Numb’s role in neuronal development causes null mutations to be lethal, so we knocked down *Drosophila* Numb (d-Numb KD) selectively in neurons using RNAi transgenes (*UAS-Numb^ds 5,10^*; Tang *et al.*, 2005). Numb RNAi was expressed when the inducer, RU-486, was added to the culture media (Berke *et al.*, 2013; Nicholson *et al.*, 2008; Osterwalder *et al.*, 2001; Roman *et al.*, 2001). The crawling behavior of these experimental animals was compared to that of control larvae reared on the same food but without RU-486. Wandering-stage larvae were identified on the sides of culture vials, washed, and gently placed on 0.5% agarose plates to crawl for 2min. Videos and photos of the crawling trajectory and searching behavior (indicated in Fig. 1A) were compared (Methods). Total distance traveled, and hence average velocity, was assessed in straight and curved paths that lacked searches. NumbKD did not alter the distance crawled [Mean±SEM(n); control: 7.52±0.64cm(29) vs Numb KD: 7.56±0.40cm(21)]. However, NumbKD significantly increased larval searching. In both groups, the number of searches per 2min trial ranged from 0-9, but 31 of 75 (41%) of Numb KD animals searched 3 or more times while only 18 of 74 (24%) of the control showed this level of searching behavior (p=0.0046). The bias towards increased searching resulted in a significantly higher proportion of irregular path types, characterized by sharp turns and short linear segments between turns (Fig. 1B, left hand pair of bars, p = 0.016).

To verify that the increase in searching behavior was due to a reduction in *Drosophila* Numb (d-Numb) expression, we attempted to rescue this defect by co-expressing an RNAi-insensitive homolog from mouse (m-Numb; Numb4; *UAS-numb4*; Tang *et al.*, 2005). Like d-Numb, the mouse isoforms contain the conserved DPF and NPF motifs, involved in membrane recycling (Santolini *et al.*, 2000), and they share significant homology within the PTB domain, known to recruit E3 ligases (Dho *et al.*, 1998; Fig 1C). m-Numb also contains a putative proline rich region (PRR) (aa 183-555; not shown in Fig. 1), which interacts with SH3 domains of partner proteins (Luo *et al.*, 2020). The mammalian isoform used for rescue of the searching phenotype (m-Numb4; Huang *et al.*, 2005) has been used previously to rescue defects in cellular differentiation and fate selection *in vivo* (Tang *et al.*, 2005; Verdi *et al.*, 1999; Zhong *et al.*, 1996). We first verified that the addition of a second UAS transgene (*UAS-mCD8-GFP*) would not diminish the increased search behavior caused by NumbKD (Fig. 1B, middle pair of bars). When m-Numb replaced GFP as the second downstream UAS transgene, the hyperactive searching behavior was significantly reduced to control levels (trajectories with 3+ searches; p=0.2307) leading to a distribution of path types similar to that seen with the control (Fig. 1B, right hand pair of bars; p = 0.90). This rescue is in line with the conserved function of Numb between flies and mice (Zhong *et al.*, 1996).

The regulation of searching behavior in *Drosophila* larvae is both complex and incompletely understood. Sensory feedback from peripheral chordotonal organs and multi-dendritic neurons can initiate searching (Ainsley *et al.*, 2003; Guo *et al.*, 2016; Ohyama *et al.*, 2013), the behavior can be stimulated by a small population of ventral ganglia interneurons (Clark *et al.*, 2016), and searching is influenced by several neurotransmitter systems (Okusawa *et al.*, 2014; Saraswati *et al.*, 2004; Selcho *et al.*, 2012; Suster *et al.*, 2004). Numb may influence one or more of these regulatory mechanisms, and more specific RNAi expression would be instructive. The inducible nature of the GeneSwitch system and the ability to rescue the searching phenotype with m-Numb will provide interesting opportunities to dissect both the relevant spatial (cellular) and developmental aspects of Numb signaling. In this regard, it is possible that Numb-dependent protein trafficking, proteosomal-dependent degradation, endocytosis, axonal/synaptic targeting, or cellular differentiation may individually or coordinately cause this behavioral phenotype. Lastly, double-mutant analysis of larval searching may be an efficient assay to identify Numb’s evolutionarily-conserved interactors.

## Methods

*Fly stocks.* All fly strains were cultured on a standard dextrose/cornmeal medium at 21ºC. *ELAV-GS-GAL4* (FlyBase ID FBti0116249, a gift from H. Keshishian, Yale University) was used to express *UAS* constructs in all neurons. GeneSwitch (GS) is a bi-partite expression system that uses a conditional RU-486 dependent GAL4 protein to induce transgene expression (Berke *et al.*, 2013; Osterwalder *et al.*, 2001). The parents/progeny of experimentals and controls developed with and without 5µg/ml RU-486 in the media, respectively. *UAS-Numb^ds5,10^* and *UAS-mNumb#4* (both gifts from W. Zhong, Yale University) were used to knockdown d-Numb and express m-Numb. *UAS-CD8-GFP* (FlyBase ID FBti0131931; Bloomington Stock Center, NIH P40OD018537) was used during the co-expression control experiments.

*Larval locomotion*. Wandering, third instar larvae were each gently removed from their culture vial, washed and sexed, and then placed in the center of 0.5% agarose plates at the start of each trial. The 2min trials began when productive crawling was observed and they were recorded using a Sony HDR-CX405 Camcorder (Sony Corporation, New York, NY). Only trials lasting the full time were used. Any trial in which larvae collided with the edge of the 14cm diameter plate were ended and not used. At the end of each trial, the animal was removed and the mouth hook imprints were photographed and used to categorize the type of trajectory. Locomotion was classified as ‘straight’ if the mouth hook imprints did not deviate from a straight line by more than 1cm from a line connecting the path’s start and end points. If so, the path was classified as ‘curved’. Any path that involved three or more searching events was classified as ‘irregular’. A ‘search’ was defined as the presence of a partially followed path (3 mouth hook imprints that were distinct from the main path) that was separated from other searches by at least three imprints. Path lengths for straight and curved paths with no searches were measured manually. Larvae were never re-tested during additional trials and animals were only taken from healthy culture vials. Over the course of the experiments, third instar larvae were taken from multiple culture vials and were the progeny of several sets of parental animals.

*Statistics.* All comparisons were examined with chi-square tests in Minitab 18 (Minitab, LLC., State College, PA). Statistical significance is indicated as p<0.05 (*), p<0.005 (**) and not significant (ns) in Fig. 1.

## References

[R1] Ainsley JA, Pettus JM, Bosenko D, Gerstein CE, Zinkevich N, Anderson MG, Adams CM, Welsh MJ, Johnson WA (2003). Enhanced locomotion caused by loss of the Drosophila DEG/ENaC protein Pickpocket1.. Curr Biol.

[R2] Berke B, Wittnam J, McNeill E, Van Vactor DL, Keshishian H (2013). Retrograde BMP signaling at the synapse: a permissive signal for synapse maturation and activity-dependent plasticity.. J Neurosci.

[R3] Clark MQ, McCumsey SJ, Lopez-Darwin S, Heckscher ES, Doe CQ (2016). Functional Genetic Screen to Identify Interneurons Governing Behaviorally Distinct Aspects of Drosophila Larval Motor Programs.. G3 (Bethesda).

[R4] Clark MQ, Zarin AA, Carreira-Rosario A, Doe CQ (2018). Neural circuits driving larval locomotion in Drosophila.. Neural Dev.

[R5] Dho SE, Jacob S, Wolting CD, French MB, Rohrschneider LR, McGlade CJ (1998). The mammalian numb phosphotyrosine-binding domain. Characterization of binding specificity and identification of a novel PDZ domain-containing numb binding protein, LNX.. J Biol Chem.

[R6] Fox LE, Soll DR, Wu CF (2006). Coordination and modulation of locomotion pattern generators in Drosophila larvae: effects of altered biogenic amine levels by the tyramine beta hydroxlyase mutation.. J Neurosci.

[R7] Guo Y, Wang Y, Zhang W, Meltzer S, Zanini D, Yu Y, Li J, Cheng T, Guo Z, Wang Q, Jacobs JS, Sharma Y, Eberl DF, Göpfert MC, Jan LY, Jan YN, Wang Z (2016). Transmembrane channel-like (tmc) gene regulates Drosophila larval locomotion.. Proc Natl Acad Sci U S A.

[R8] Heckscher ES, Lockery SR, Doe CQ (2012). Characterization of Drosophila larval crawling at the level of organism, segment, and somatic body wall musculature.. J Neurosci.

[R9] Huang EJ, Li H, Tang AA, Wiggins AK, Neve RL, Zhong W, Jan LY, Jan YN (2004). Targeted deletion of numb and numblike in sensory neurons reveals their essential functions in axon arborization.. Genes Dev.

[R10] Hughes CL, Thomas JB (2007). A sensory feedback circuit coordinates muscle activity in Drosophila.. Mol Cell Neurosci.

[R11] Luo Z, Mu L, Zheng Y, Shen W, Li J, Xu L, Zhong B, Liu Y, Zhou Y (2020). NUMB enhances Notch signaling by repressing ubiquitination of NOTCH1 intracellular domain.. J Mol Cell Biol.

[R12] Nicholson L, Singh GK, Osterwalder T, Roman GW, Davis RL, Keshishian H (2008). Spatial and temporal control of gene expression in Drosophila using the inducible GeneSwitch GAL4 system. I. Screen for larval nervous system drivers.. Genetics.

[R13] Ohyama T, Jovanic T, Denisov G, Dang TC, Hoffmann D, Kerr RA, Zlatic M (2013). High-throughput analysis of stimulus-evoked behaviors in Drosophila larva reveals multiple modality-specific escape strategies.. PLoS One.

[R14] Okusawa S, Kohsaka H, Nose A (2014). Serotonin and downstream leucokinin neurons modulate larval turning behavior in Drosophila.. J Neurosci.

[R15] Osterwalder T, Yoon KS, White BH, Keshishian H (2001). A conditional tissue-specific transgene expression system using inducible GAL4.. Proc Natl Acad Sci U S A.

[R16] Pece S, Confalonieri S, R Romano P, Di Fiore PP (2010). NUMB-ing down cancer by more than just a NOTCH.. Biochim Biophys Acta.

[R17] Roman G, Endo K, Zong L, Davis RL (2001). P[Switch], a system for spatial and temporal control of gene expression in Drosophila melanogaster.. Proc Natl Acad Sci U S A.

[R18] Santolini E, Puri C, Salcini AE, Gagliani MC, Pelicci PG, Tacchetti C, Di Fiore PP (2000). Numb is an endocytic protein.. J Cell Biol.

[R19] Saraswati S, Fox LE, Soll DR, Wu CF (2004). Tyramine and octopamine have opposite effects on the locomotion of Drosophila larvae.. J Neurobiol.

[R20] Selcho M, Pauls D, El Jundi B, Stocker RF, Thum AS (2012). The role of octopamine and tyramine in Drosophila larval locomotion.. J Comp Neurol.

[R21] Song W, Onishi M, Jan LY, Jan YN (2007). Peripheral multidendritic sensory neurons are necessary for rhythmic locomotion behavior in Drosophila larvae.. Proc Natl Acad Sci U S A.

[R22] Spana EP, Kopczynski C, Goodman CS, Doe CQ (1995). Asymmetric localization of numb autonomously determines sibling neuron identity in the Drosophila CNS.. Development.

[R23] Suster ML, Karunanithi S, Atwood HL, Sokolowski MB (2004). Turning behavior in Drosophila larvae: a role for the small scribbler transcript.. Genes Brain Behav.

[R24] Tang H, Rompani SB, Atkins JB, Zhou Y, Osterwalder T, Zhong W (2005). Numb proteins specify asymmetric cell fates via an endocytosis- and proteasome-independent pathway.. Mol Cell Biol.

[R25] Uemura T, Shepherd S, Ackerman L, Jan LY, Jan YN (1989). numb, a gene required in determination of cell fate during sensory organ formation in Drosophila embryos.. Cell.

[R26] Verdi JM, Bashirullah A, Goldhawk DE, Kubu CJ, Jamali M, Meakin SO, Lipshitz HD (1999). Distinct human NUMB isoforms regulate differentiation vs. proliferation in the neuronal lineage.. Proc Natl Acad Sci U S A.

[R27] Vervoort M, Merritt DJ, Ghysen A, Dambly-Chaudière C (1997). Genetic basis of the formation and identity of type I and type II neurons in Drosophila embryos.. Development.

[R28] Wang JW, Sylwester AW, Reed D, Wu DA, Soll DR, Wu CF (1997). Morphometric description of the wandering behavior in Drosophila larvae: aberrant locomotion in Na+ and K+ channel mutants revealed by computer-assisted motion analysis.. J Neurogenet.

[R29] Wang JW, Soll DR, Wu CF (1970). Morphometric description of the wandering behavior in Drosophila larvae: a phenotypic analysis of K+ channel mutants.. J Neurogenet.

[R30] Wang N, Wang DD, Shen Y (2019). Numb deficiency causes impaired trafficking of mGlu5 in neurons and autistic-like behaviors.. Neurosci Lett.

[R31] Zhong W, Feder JN, Jiang MM, Jan LY, Jan YN (1996). Asymmetric localization of a mammalian numb homolog during mouse cortical neurogenesis.. Neuron.

[R32] Zhou P, Alfaro J, Chang EH, Zhao X, Porcionatto M, Segal RA (2011). Numb links extracellular cues to intracellular polarity machinery to promote chemotaxis.. Dev Cell.

[R33] Zhou L, Yang D, Wang DJ, Xie YJ, Zhou JH, Zhou L, Huang H, Han S, Shao CY, Li HS, Zhu JJ, Qiu MS, De Zeeuw CI, Shen Y (2015). Numb deficiency in cerebellar Purkinje cells impairs synaptic expression of metabotropic glutamate receptor and motor coordination.. Proc Natl Acad Sci U S A.

